# Supplementary material for: Noradrenergic neuromodulation produces a NMDAR-dependent network state of respiratory rhythmogenesis in the preBötzinger Complex
Source: bioRxiv. 2026 Feb 13:2026.02.11.705209. Preprint. [Version 1] doi: 10.64898/2026.02.11.705209 (PMC12919053; doi:10.64898/2026.02.11.705209)
Supplement: Supplement 1 [file media-1.pdf]

Supplementary Materials: Noradrenergic neuromodulation produces a NMDAR-dependent network state of respiratory rhythmogenesis in the preBötzinger Complex

Andrew Kieran Tryba, Jean-Charles Viemari, Yangyang Wang, Alfredo J. Garcia, 3rd

1 Model description

The model describes a network of $N = 100$ synaptically coupled excitatory neurons. For each preBötC neuron, we consider a single-compartment model neuron that incorporates Hodgkin-Huxley-style conductances adapated from previously described models (Jasinski et al., 2013; Wang and Rubin, 2017; Phillips et al., 2019; Phillips and Rubin, 2022). Each neuron's dynamics are governed by the following differential equations:

$$\begin{aligned}
C\frac{dV}{dt} &= -I_{\mathrm{Na}} - I_{\mathrm{NaP}} - I_{\mathrm{K}} - I_{\mathrm{Ca}} - I_{\mathrm{CAN}} - I_{\mathrm{L}} - I_{\mathrm{NMDA}} - I_{\mathrm{nonNMDA}} - I_{\mathrm{Tonic}} \\
\frac{dy}{dt} &= (y_{\infty}(V) - y)/\tau_y(V), \quad y = \{m_{\mathrm{Na}}, h_{\mathrm{Na}}, m_{\mathrm{Nap}}, h_{\mathrm{Nap}}, m_{\mathrm{Ca}}, h_{\mathrm{Ca}}, m_{\mathrm{K}}\} \\
\frac{d\mathrm{Ca_{tot}}}{dt} &= -\alpha_{\mathrm{Ca}}(P_{\mathrm{ICa}} I_{\mathrm{Ca}} + P_{\mathrm{nonNMDAca}} \cdot I_{\mathrm{nonNMDA}} + P_{\mathrm{NMDAca}} \cdot I_{\mathrm{NMDA}}) \\
&\quad -(\mathrm{Ca_i} - \mathrm{Ca_{min}})/\tau_{\mathrm{Ca}} \\
\frac{d\mathrm{Ca_i}}{dt} &= -\alpha_{\mathrm{Ca}}(P_{\mathrm{ICa}} I_{\mathrm{Ca}} + P_{\mathrm{nonNMDAca}} \cdot I_{\mathrm{nonNMDA}} + P_{\mathrm{NMDAca}} \cdot I_{\mathrm{NMDA}}) \\
&\quad +\alpha_{\mathrm{ER}}(J_{\mathrm{ER_{IN}}} - J_{\mathrm{ER_{OUT}}}) - (\mathrm{Ca_i} - \mathrm{Ca_{min}})/\tau_{\mathrm{Ca}} \\
\frac{dl}{dt} &= AK_{\mathrm{d}}(1-l) - A\mathrm{Ca_i} l
\end{aligned} \tag{1.1}$$

The neuronal membrane potential (V) is governed by a set of membrane ionic currents. $C = 36\,\mathrm{pF}$ is neuronal membrane capacitance and t is time. Each I_i represents a current with i denoting the current's type. The ionic currents in the model include fast Na^+ current (I_{Na}), persistent Na^+ current (I_{NaP}), delayed rectifier K^+ current (I_{K}), high-voltage-activated Ca^{2+} current (I_{Ca}), Ca^{2+}-activated nonspecific cation current (I_{CAN}), K^+-dominated leak current (I_{L}), excitatory synaptic currents (I_{nonNMDA} and I_{NMDA}). I_{Tonic} denotes a fixed or tonic excitatory synaptic input (e.g., from respiratory control areas outside of the preBotC). The currents are defined in Table 1.

In Table 1, g_i is the maximum conductance. Activation (m_i) and inactivation (h_i) variables for most ionic channels I_i are governed by the second y equation in (1.1), where steady-state activation (m_{∞}) and inactivation (h_{∞}) functions and time constants are described as in Table 2. Notice from the bottom row in Table 2 that unlike the other currents, I_{CAN} has

Table 1: Ionic currents and channel reversal potentials for system (1.1)

Currents (pA)	Reversal potentials (mV)
$I_{\text{Na}} = g_{\text{Na}} m_{\text{Na}}^3 h_{\text{Na}} (V - E_{\text{Na}})$<br>$I_{\text{NaP}} = g_{\text{NaP}} m_{\text{NaP}} h_{\text{NaP}} (V - E_{\text{Na}})$	$E_{\text{Na}} = 26.54 \ln(Na_o/Na_i)$
$I_{\text{K}} = g_{\text{K}} m_{\text{K}}^4 (V - E_{\text{K}})$	$E_{\text{K}} = 26.54 \ln(K_o/K_i)$
$I_{\text{Ca}} = g_{\text{Ca}} m_{\text{Ca}} h_{\text{Ca}} (V - E_{\text{Ca}})$	$E_{\text{Ca}} = 13.27 \ln(\text{Ca}_\text{o}/\text{Ca}_\text{i})$
$I_{\text{CAN}} = g_{\text{CAN}} m_{\text{CAN}} (V - E_{\text{CAN}})$	$E_{\text{CAN}} = 0$
$I_{\text{L}} = g_{\text{L}} (V - E_{\text{L}})$	$E_{\text{L}} = -26.54 \ln((Na_i + 42K_i)/(Na_o + 42K_o))$
$I_{\text{nonNMDA}} = g_{\text{nonNMDA}} (V - E_{\text{SynE}})$<br>$I_{\text{NMDA}} = g_{\text{NMDA}} B(V) (V - E_{\text{SynE}})$<br>$I_{\text{Tonic}} = g_{\text{Tonic}} (V - E_{\text{SynE}})$	$E_{\text{SynE}} = 0$

instantaneous activation, which depends on Ca_i and is voltage-independent. The parameters for these currents are specified in Table 3.

Table 2: Functions associated with activation and inactivation variables for system (1.1). We use the variable y when an expression corresponds to a set of variables.

gating variables	steady state activation and inactivation	time constants
m_{Na}	$y_\infty(V) = 1/(1 + \exp(-(V - V_{y1/2})/k_y))$	$\tau_y(V) = \tau_{y\max}/\cosh(-(V - V_{\tau y1/2})/k_{\tau y})$
h_{Na}		
m_{NaP}		
h_{Na}		
m_{Ca}		$\tau_{m_{\text{Ca}}} = 0.5\,\text{ms}$
h_{Ca}		$\tau_{h_{\text{Ca}}} = 18\,\text{ms}$
m_{K}	$m_{\text{K}\infty} = \alpha_\infty/(\alpha_\infty + \beta_\infty)$	$\tau_{m_{\text{K}}} = 1/(\alpha_\infty + \beta_\infty)$
	$\alpha_\infty = A_\alpha \cdot (V + B_\alpha)/(1 - \exp(-(V + B_\alpha)/k_\alpha))$, $\beta_\infty = A_\beta \cdot \exp(-(V + B_\beta)/k_\beta)$	
m_{CAN}	$m_{\text{CAN}} = 1/(1 + (K_{\text{CAN}}/\text{Ca}_\text{i})^n)$	
Magnesium block	$B(V) = 1/(1 + e^{-0.062V}[\text{Mg}^{2+}]/3.57)$	

The dynamics of the total intracellular Ca^{2+} concentration within the cell (Ca_{tot}) and intracellular concentration of free Ca^{2+} (Ca_i) are described by the third and fourth equations in (1.1), respectively. Both variables are intimately linked with l, the fraction of IP_3 receptors that are not inactivated by calcium, which is governed by the last equation of (1.1). In the calcium equations, J_{ERin} represents the flux of Ca^{2+} per unit volume from the endoplasmic reticulum (ER) into the cytoplasm, which depends on l, and J_{ERout} represents the flux of Ca^{2+} per unit volume from the cytoplasm into the ER. These two fluxes are modeled by (1.2):

Table 3: Parameter values for system (1.1)

Current	Parameters
Fast Na^+ (I_{Na})	$g_{Na} = 150\,nS$, $Na_i = 15$ mM, $Na_o = 120$ mM
	$V_{m1/2} = -43.8\,mV, k_m = 6\,mV, \tau_{mmax} = 0.25\,ms,$ $V_{\tau m1/2} = -43.8\,mV, k_{\tau m} = 14\,mV$ $V_{h1/2} = -67.5\,mV, k_h = -11.8\,mV$ $\tau_{hmax} = 8.46\,ms$, $V_{\tau h1/2} = -67.5\,mV, k_{\tau h} = 12.8\,mV$
Persistent Na^+ (I_{NaP})	$g_{NaP} = N(\mu_{NaP}, \sigma_{NaP})\,nS$, $\mu_{NaP} = 3.33\,nS$ $\sigma_{NaP} = 0.75\,nS$
	$V_{m1/2} = -47.1\,mV, k_m = 3.1\,mV, \tau_{mmax} = 1\,ms,$ $V_{\tau m1/2} = -47.1\,mV, k_{\tau m} = 6.2\,mV$ $V_{h1/2} = -60\,mV, k_h = -9\,mV, \tau_{hmax} = 5000\,ms,$ $V_{\tau h1/2} = -60\,mV, k_{\tau h} = 9\,mV$
K^+ delayed rectifier (I_K)	$g_K = 220\,nS$, $K_o = 6.5$ mM, $K_i = 125$ mM
	$A_\alpha = 0.01, B_\alpha = 44\,mV, k_\alpha = 5\,mV, A_\beta = 0.17,$ $B_\beta = 49\,mV, k_\beta = 40\,mV$
Ca^{2+} (I_{Ca})	$g_{Ca} = 0.000065\,nS$, $Ca_o = 4$ mM
	$V_{m1/2} = -27.5\,mV, k_m = 5.7\,mV$ $V_{h1/2} = -52.4\,mV, k_h = -5.2\,mV$
Ca^{2+}-activated nonspecific (I_{CAN})	$g_{CAN} = VAR, K_{CAN} = 0.00074\,mM, n = 0.97$
Leakage (I_L)	$g_L = N(\mu_L, \sigma_L)$, $\mu_L = exp((K_o - 3.425)/4.05)\,nS$ $\sigma_L = 0.05 \cdot \mu_L\,nS$
non-NMDA synaptic ($I_{nonNMDA}$)	$g_{nonNMDA}$ = VAR, see (1.3), $\tau_{nonNMDA} = 5\,ms$
NMDA synaptic (I_{NMDA})	g_{NMDA} = VAR, see (1.3), $\tau_{NMDA} = 20\,ms$, $[Mg^{2+}] = 2\,mM$
Tonic synaptic (I_{Tonic})	$g_{Tonic} = 0.3\,nS$

$$J_{ER_{IN}} = \left(L_{IP_3} + P_{IP_3} \left[\frac{[IP_3]Ca_i l}{([IP_3] + K_I)(Ca_i + K_a)} \right]^3 \right) (Ca_{ER} - Ca_i) \tag{1.2a}$$

$$J_{ER_{OUT}} = V_{SERCA} \frac{Ca_i^2}{K_{SERCA}^2 + Ca_i^2} \tag{1.2b}$$

$$Ca_{ER} = \frac{Ca_{tot} - Ca_i}{\sigma}. \tag{1.2c}$$

The values of parameters associated with the equations for Ca_{tot}, Ca_i and l are given by: $\alpha_{Ca} = 2.5 \times 10^{-5}$ mM/fC, $\tau_{Ca} = 500$ ms, $Ca_{min} = 5.0 \times 10^{-6}$ mM, $\alpha_{ER} = 2.5 \times 10^{-5}$, $L_{IP3} = 0.1$/ms, $P_{IP_3} = 77500$/ms, $[IP_3] = 1.5 \times 10^{-3}$ mM, $K_I = 10^{-3}$ mM, $K_a = 10^{-4}$ mM, $V_{SERCA} = 0.45$ mM/ms, $K_{SERCA} = 7.5 \times 10^{-5}$ mM, $\sigma = 0.185$, $A = 0.1$ mM/ms and $K_d = 0.2 \times 10^{-3}$ mM. Additional description of model components has been given previously Toporikova and Butera (2011); Park and Rubin (2013); Jasinski et al. (2013); Wang and Rubin (2017); Phillips and Rubin (2022).

In addition, there are various sources of Ca^{2+} influx from the extracellular space: voltage-gated Ca^{2+} channels (I_{Ca}) and synaptic inputs where a percentage of the synaptic current is assumed to consist of Ca^{2+} ions. The specific mechanisms behind the synaptic calcium sources are unclear and likely depend on specific types of glutamate receptors. There are three subtypes of ionotropic glutamate receptors, N-methyl-D-aspartate (NMDA), Kainate (KAR), and a-amino-3-hydroxy-5-methyl-4-isoxazolepropionic acid (AMPA), all of which are expressed in the pre-BötC and have varying degrees of calcium permeability. Among them, AMPA is not permeable to Ca^{2+}, whereas KAR and NMDA are possible candidates for synaptically mediated calcium entry (Phillips et al., 2019; Morgado-Valle and Feldman, 2007; Ermentrout and Terman, 2010). As a result, we include three possible sources for calcium influx in the $\mathrm{Ca_i}$ and $\mathrm{Ca_{tot}}$ equations (see (1.1)): $P_{\mathrm{ICa}} I_{\mathrm{Ca}}$, $P_{\mathrm{nonNMDAca}} \cdot I_{\mathrm{nonNMDA}}$ and $P_{\mathrm{NMDAca}} \cdot I_{\mathrm{NMDA}}$. The parameters P_{ICa}, $P_{\mathrm{nonNMDAca}}$ and P_{NMDAca} control the relative calcium permeability from each source. Following (Phillips et al., 2019), we denote the model with $P_{\mathrm{nonNMDAca}} = P_{\mathrm{NMDAca}} = 0$ as the CaV model, the model with $P_{\mathrm{ICa}} = P_{\mathrm{NMDAca}} = 0$ as the CaK model, and the model with $P_{\mathrm{ICa}} = P_{\mathrm{nonNMDAca}} = 0$ as the CaN model.

In (1.1), the synaptic conductance of fast AMPA/kainate synapse (denoted as g^i_{nonNMDA}) and NMDA receptors (g^i_{NMDA}) for the i-th neuron in the population are described by the following equations:

$$\begin{aligned} g^i_{\mathrm{nonNMDA}} &= \textstyle\sum_{j \neq i, n} W_{\mathrm{nonNMDA},ji} D_j C_{j,i} H(t - t_{j,n}) e^{-(t - t_{j,n})/\tau_{\mathrm{nonNMDA}}} \\ g^i_{\mathrm{NMDA}} &= \textstyle\sum_{j \neq i, n} W_{\mathrm{NMDA},ji} D_j C_{ji} H(t - t_{j,n}) e^{-(t - t_{j,n})/\tau_{\mathrm{NMDA}}} \end{aligned} \tag{1.3}$$

where $W_{\mathrm{nonNMDA},ji}$ and $W_{\mathrm{NMDA},ji}$ represent the weights of the non-NMDA and NMDA synaptic connection from neuron j to neuron i. D_j is a scaling factor for short-term synaptic depression in the presynaptic neuron j. It evolves according to the equation $\frac{dD_j}{dt} = \frac{D_0 - D_j}{\tau_D} - \alpha_D D_j \delta(t - t_j)$, where $D_0 = 1$, $\tau_D = 1000\,\mathrm{ms}$, $\alpha_D = 0.2$ and $\delta(.)$ is the Kronecker delta function that equals 1 at the time of each spike in neuron j and 0 otherwise. Additional description of the synaptic depression and related parameters have been given in (Phillips and Rubin, 2022). $C_{j,i}$ is an element of the connectivity matrix ($C_{j,i} = 1$ if neuron j makes a synapse with neuron i and $C_{j,i} = 0$ otherwise), $H(.)$ is the Heaviside step function, and t denotes time. τ_{nonNMDA} and τ_{NMDA} are exponential synaptic decay constants for non-NMDA and NMDA receptors (see Table 3). $t_{j,n}$ is the time at which the n-th action potential generated by neuron j reaches neuron i.

The current for an NMDA synapse is modeled differently from that of AMPA/kainate (see Table 1) because, under normal physiological conditions, the NMDA receptor is partially blocked by magnesium ions (Ermentrout and Terman, 2010). Specificically, $B(V)$, as defined in the last row of Table 2 within the I_{NMDA} function in Table 1, represents this magnesium block. As discussed above, these currents contribute to synaptically triggered Ca^{2+} transients (see (1.1)), where $P_{\mathrm{nonNMDAca}}$ and P_{NMDAca}) denote the percentages of Ca^{2+} ions that can pass through AMPA/kainate (I_{nonNMDA}) and NMDA synaptic currents (I_{NMDA}), respectively. In our simulations, the calcium permeability parameters were varied over the range $P_{\mathrm{nonNMDAca}} \in [0, 0.0015]$ and $P_{\mathrm{NMDAca}} \in [0, 0.005]$. Excitatory synaptic weights were drawn from a uniform distribution, $W_{j,i} = U(0, W_{\mathrm{Max}})$, with $W_{\mathrm{Max}} = 0.15\,\mathrm{nS}$ for non-NMDA

receptors and $W_{\text{Max}} = 0.05\,\text{nS}$ for NMDA receptors. The higher range for P_{NMDAca} reflects the relatively higher calcium permeability of NMDARs, whereas calcium influx through non-NMDA receptors primarily arises from KARs Phillips et al. (2019); Paarmann et al. (2000); Perrais et al. (2009). In contrast, the smaller maximum NMDAR synaptic weight reflects experimental findings that, under normal conditions, NMDAR-mediated coupling is significantly weaker than that of AMPARs in the preBotC (Morgado-Valle and Feldman, 2007). In addition, g_{CAN} was uniformly distributed over the range $[0, \bar{g}_{\text{CAN}}]$ nS. The elements of the network connectivity matrix, $C_{j,i}$, are randomly assigned values of 0 or 1 such that the probability of any connection between neuron j and neuron i being 1 is equal to the network connection probability $P_{\text{Syn}} = 0.13$. We examine the effects of NMDA synaptic strength and I_{CAN} on the dynamcis of our model by varying $W_{\text{Max,NMDA}}$ and $\bar{g}_{\text{CAN}}$ (see, e.g., supplentary Figure 2).
