# Supplementary material for: Noradrenergic neuromodulation produces a NMDAR-dependent network state of respiratory rhythmogenesis in the preBötzinger Complex
Source: bioRxiv. 2026 Feb 13:2026.02.11.705209. Preprint. [Version 1] doi: 10.64898/2026.02.11.705209 (PMC12919053; doi:10.64898/2026.02.11.705209)
Supplement: Supplement 2 [file NIHPP2026.02.11.705209v1-supplement-2.pdf]

bioRxiv preprint doi: https://doi.org/10.64898/2026.02.11.705209; this version posted February 14, 2026. The copyright holder for this preprint (which was not certified by peer review) is the author/funder, who has granted bioRxiv a license to display the preprint in perpetuity. It is made available under aCC-BY 4.0 International license.

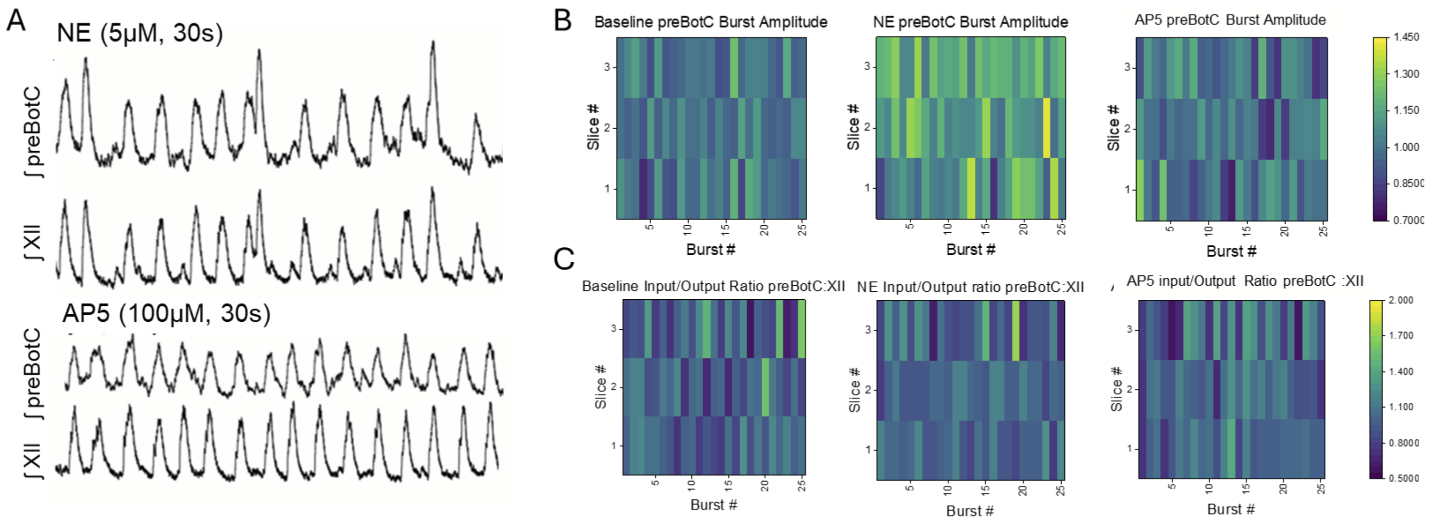

Supplemental Figure 1. **After NE modulation, subsequently blocking NMDAR does not result in loss of the ability of the preBötC to faithfully drive bursting in the hypoglossal (XII) motor pool. (Suppl. A)** Raw data and **(Suppl. B)** heatmaps illustrating that compared to baseline (ACSF), following bath-application of 5uM NE, integrated preBötC network amplitude (p=0.027 preBötC, One-Sample T-test) and frequency increases (p=0.029, n=3 preBötC; also see Figs 1&2). **(Suppl. A&C)** Following NE-modulation, subsequent blockade of NMDAR with APV does not prevent transmission of preBötC bursting to trigger XII motor output. Heatmap illustrates the input-output relationship between preBötC activity and XII motor output. Heatmaps include n=3 slice preparations (slices # 1-3, y-axis) and 25 consecutive bursts (burst #1-25, x-axis).

bioRxiv preprint doi: https://doi.org/10.64898/2026.02.11.705209; this version posted February 14, 2026. The copyright holder for this preprint (which was not certified by peer review) is the author/funder, who has granted bioRxiv a license to display the preprint in perpetuity. It is made available under aCC-BY 4.0 International license.

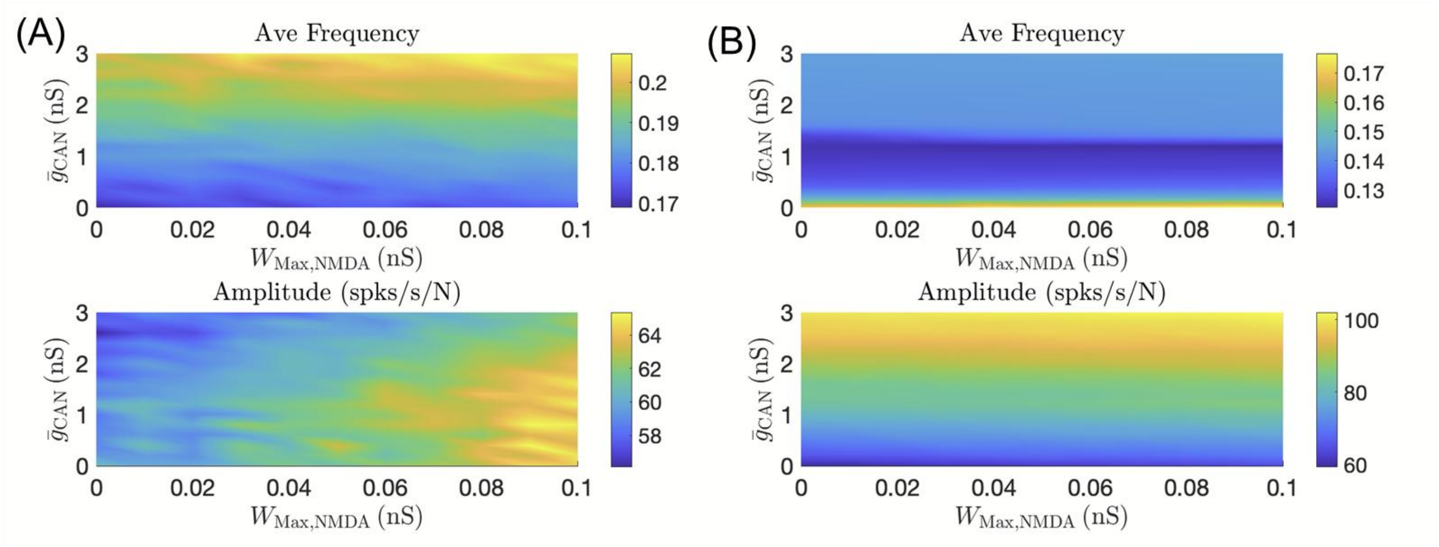

Supplementary Figure 2. Effects of varying CAN conductance (g̅_CAN) and NMDAR weight (W_Max ,_NMDA) on the dynamics of **(A)** the CaV network where calcium influx is sourced only from voltage-gated calcium channel currents, and **(B)** the CaK network where calcium influx is exclusively from non-NMDA synaptic currents. In both models, for a fixed g̅_CAN, varryng NMDAR weight has minimal impact on network frequency and amplitude (note the small scales on the color bar for the bottom figure in **(A)**).
